# Associations of pain severity and mobility with age in chronic low back pain: does the type of assessment matter?

**DOI:** 10.1093/geroni/igab046.3224

**Published:** 2021-12-17

**Authors:** Taylor Buchanan, Deanna Rumble, Kristen Allen-Watts, Katie O'Neal, Tammie Quinn, Thomas Buford, Burel Goodin

**Affiliations:** University of Alabama at Birmingham, Birmingham, Alabama, United States

## Abstract

Chronic low back pain (cLBP) can lead to severe pain symptoms as well as disability in adults. As individuals age, pain symptoms and mobility outcomes can become increasingly debilitating. However, current findings regarding the influence of age on symptoms and outcomes are mixed and may be attributed to the assessment methodologies for pain and mobility. Therefore, we sought to examine the association of age with broad and specific assessments of pain severity and mobility commonly implemented in adults with cLBP. cLBP participants (n = 158) completed questionnaires regarding pain intensity and disability including demographics, Clinical Pain Assessment (CPA) and the Oswestry Low Back Pain questionnaire (OLBP). Participants also completed assessments of movement-evoked pain and difficulty by performing the Short Physical Performance Battery (SPPB). Pearson’s chi-square tests and regression-based analyses were conducted using SPSS version 26.0. Among cLBP participants, age was associated with pain-related disability indexed by section one of the OLBPS regarding pain intensity (F= 5.0, p<.05), and mobility via total SPPB score (F= 11.7, p<.05). Interestingly, age predicted greater self-reported difficulty climbing stairs (F= 21.7, p<.05), performing chores (F= 17.0, p<.05), walking (F= 14.0, p<.05), and running errands (F= 13.4, p<.05) from the CPA. Further, age predicted total balance (F= 3.2, p<.05), gait speed (F= 7.8, p<.05), and chair stand (F= 6.5, p<.05) scores of SPPB. Age is associated with questionnaires assessing cLBP pain severity and is also associated with mobility outcomes. Future research should seek to understand the influence of age on movement-evoked pain in cLBP.

